# Biomolecular Insights Into the Antifungal Activity of *Allium* Species Constituents: From Mechanisms to Applications

**DOI:** 10.5812/ijpr-162031

**Published:** 2025-12-13

**Authors:** Saeid Eslami, Behzad Zolfaghari, Masoud Sadeghi Dinani, Mustafa Ghanadian

**Affiliations:** 1 Department of Pharmacognosy, School of Pharmacy, Isfahan University of Medical Sciences, Isfahan, Iran; 2 Department of Pharmacognosy and Phytochemistry, Pharmaceutical Sciences Research Center, Isfahan University of Medical Sciences, Isfahan, Iran

**Keywords:** *Allium*, Antifungal Agents, Sulfur Compounds, Steroidal Saponins, Fungal Drug Resistance, Agriculture Application, Food Preservation

## Abstract

**Context:**

This review explores the antifungal potential of *Allium* species, emphasizing pure compounds identified through phytochemical studies. It also analyzes the mechanisms and efficacy of *Allium*-derived antifungal agents within pharmaceutical, agricultural, and food science applications.

**Objectives:**

To assess the antifungal properties of major *Allium* species and their bioactive compounds, and to evaluate their mechanisms of action and effectiveness across pharmaceutical treatments, agricultural pathogen control, and food preservation.

**Data Sources:**

A comprehensive literature search was conducted using major scientific databases, including Web of Science, PubMed, ScienceDirect, Scopus, and Google Scholar.

**Study Selection:**

Studies reporting antifungal activities of major *Allium* species and their isolated compounds were selected based on PRISMA guidelines.

**Data Extraction:**

Data were extracted from recent research focusing on the antifungal effects, mechanisms of action, and minimum inhibitory concentrations of sulfur compounds and saponins derived from *Allium* species.

**Results:**

Sulfur-containing compounds such as allicin and ajoene were found to disrupt fungal cell metabolism, destabilize cellular structures, and induce oxidative stress. These compounds showed strong activity against pathogens including *Candida albicans* and *Aspergillus fumigatus*. Saponins were also identified as key antifungal agents, with spirostane and spirostanol saponins from species like *A. ampeloprasum* and *A. porrum* demonstrating activity against *C. albicans*, *A. niger*, and *Fusarium culmorum*. Additional saponins — such as Fistoloside C, Minutoside B, and Ceposide variants — exhibited promising antifungal potential, particularly in combination therapies. Reported minimum inhibitory concentrations ranged from 0.15 µg/mL for sulfur compounds to 3.1–800+ µg/mL for saponins.

**Conclusions:**

Saponins from *Allium* species represent promising adjuncts for overcoming antifungal drug resistance and may expand treatment options beyond traditional sulfur-derived compounds. These bioactive molecules also show potential for agricultural use against soil-borne pathogens like *F. oxysporum*, as well as food preservation applications against spoilage fungi such as *Penicillium italicum* and *A. niger*. Overall, *Allium* species constitute a valuable natural source of antifungal agents with broad pharmaceutical and agricultural relevance.

## 1. Context

Fungi are a diverse group of eukaryotic organisms estimated to have over two million species. They impact global health, ecosystems, agriculture, and biomedical investigations. Additionally, fungi sometimes assemble with bacteria, which is recognized in animal research but usually disregarded in human research (1, 2). Fungi can play an indispensable role in the environment by breaking down dead organic matter and returning nutrients to the soil, which are essential for herbal growth and photosynthesis. Moreover, they have a crucial role in food production, especially in the fermentation process for making bread and cheese (3, 4).

While fungi play a positive role in ecosystems and food production, some environmental fungi can possess adaptive mechanisms, such as enzymatic abilities, that can make them opportunistic pathogens. Some of them can be harmful to plants by causing infection of the herbage and crops. Fungal spot, leaf blight, and fusarium wilt are major plant diseases (5, 6). *Fusarium oxysporum* is a widespread soil-borne fungus that causes fusarium wilt, which attacks the plant’s roots by spreading through their vascular systems and affects many crops, including potatoes, tomatoes, and beans. It disrupts the uptake of essential elements, resulting in wilting, stunted growth, and economic losses for farmers (7). Zoophilic fungi are transmitted between animals and can infect animals with their toxins, while zoonotic fungi pose risks due to close human-pet contact. This is significant because many people have pets in their homes today. Mycotoxins are secondary metabolites produced by many fungi, such as *Aspergillus* species, that contaminate animal feeds and pose public health risks. Fungi can adapt to changing environments, leading to new strains that affect both humans and animals (8-10).

There are five classes of antifungal drugs: Polyenes, echinocandins, allylamines, pyrimidine analogs, and azole derivatives (11). The widespread use of antifungal drugs leads to fungal resistance, which is a global challenge. However, the recent developments in pharmaceuticals show a hopeful outlook for the future. For example, *Allium*-derived compounds like allicin, ajoene, and other sulfur compounds have demonstrated potent antifungal activity in preclinical studies by disrupting the fungal cell membrane (12, 13).

Medicinal plants have been utilized to treat diseases for many years, and phytochemicals play a role in drug discovery and the development of bioactive compounds. Also, combining them with conventional drugs presents a viable alternative to standard therapies and can help enhance patient tolerance to antifungal agents (14). This review examines antifungal compounds derived from *Allium* species, focusing on biomolecular studies.

## 2. Methodology

This study follows the preferred reporting items for systematic reviews and meta-analyses (PRISMA) procedures, version (15), to secure a well-organized evaluation of existing research on the chemical composition and antifungal properties of *Allium* species. A thorough exploration of databases, including Web of Science, PubMed, ScienceDirect, and Google Scholar, was performed, and the literature search included Embase and CAB Abstracts to ensure comprehensive coverage of biomedical and agricultural studies related to *Allium*-derived pure compounds with antifungal effects. This search covered publications from the earliest phytochemical study on *Allium* species, 1944 (16) to 2024, using keywords such as "*Allium*"[MeSH Terms] OR garlic[Title/Abstract] OR onion[Title/Abstract]) AND (antifungal[Title/Abstract] OR fungistatic[Title/Abstract]”.

**Figure 1.**
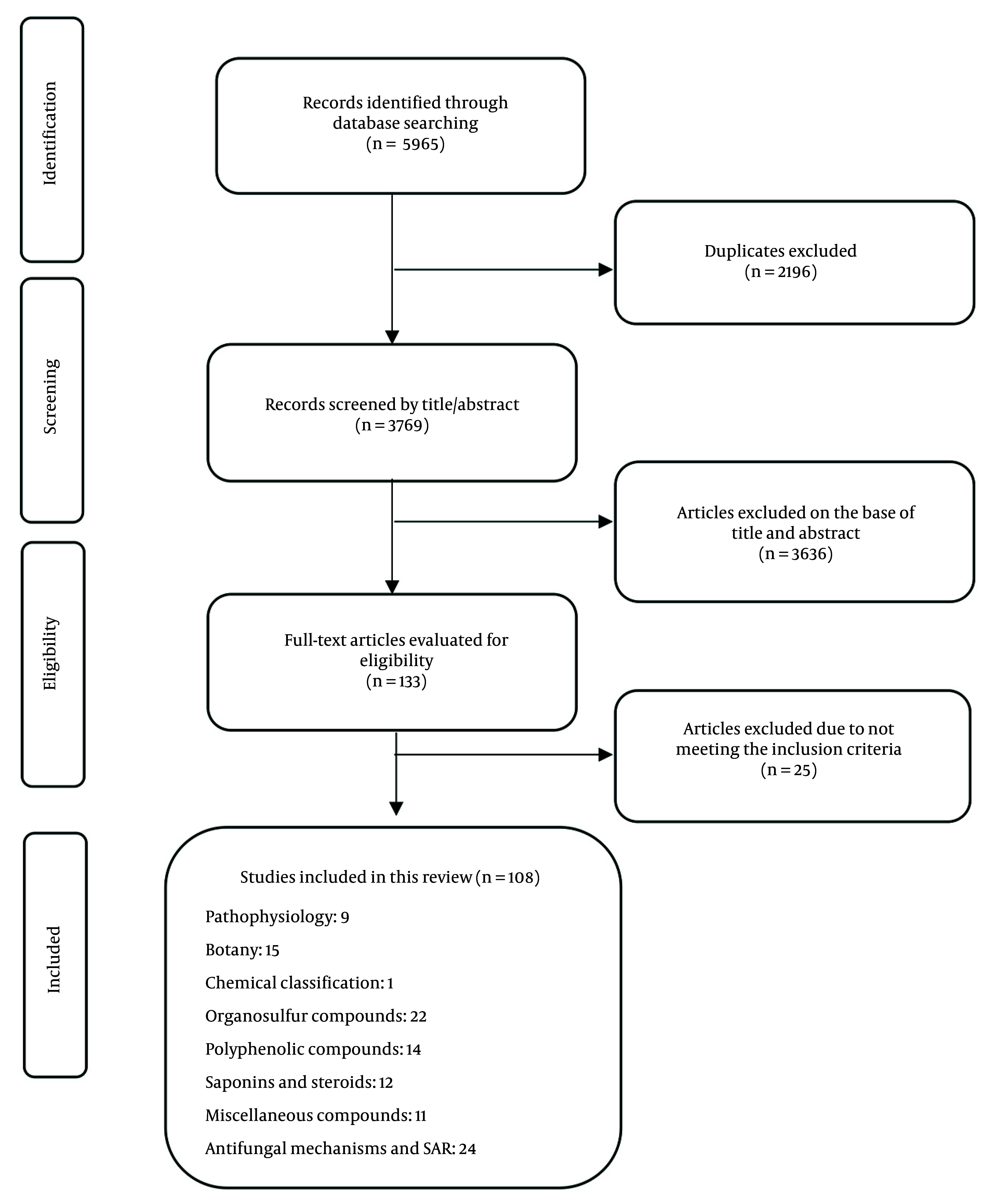
Flow diagram of study screening and selection process

This review included investigations with specific criteria to ensure quality, focusing on *Allium* species such as garlic and onion and their antifungal pure compounds: Sulfur, steroidal saponins, and phenolic compounds (Figure 1). 

Only original English-language research papers featuring in vitro experiments and isolated compounds were chosen, excluding reviews, non-English studies, and those lacking a clear antifungal or chemical description. The methodological quality of the included studies was assessed using an adjusted version of the Newcastle-Ottawa Scale tailored for in vitro research. The evaluation criteria included clarity of experimental design, reproducibility, control use, and reporting of compound purity. To minimize bias and ensure consistency, two reviewers independently screened studies and extracted data. Articles from the initial search were imported into reference management software (e.g., EndNote), where duplicate entries were removed.

## 3. Pathophysiology of Fungal Infections and Mechanism of Action

Fungi significantly impact human health, infecting billions and causing over 1.5 million deaths each year (17). They affect many human organs, such as the lungs, urinary tract, brain, and skin (18). The rise in immune system disorders, such as HIV/AIDS and autoimmune diseases, along with more organ transplants, has made many individuals more vulnerable to fungal infections (19). Another way is the pulmonary tract; fungi primarily invade the body through inhalation of spores or yeast. They often employ a "Trojan horse" mechanism, where they are carried by immune cells known as phagocytes. Additionally, fungi can enter the body through breaches in barriers or by moving through cells (transcellular) and between cells (paracellular). Initial infections typically start in the lungs; however, complications can arise, such as meningoencephalitis, when fungi cross the blood-brain barrier (e.g., *Cryptococcus neoformans* meningitis) (20).

Fungi display diverse morphological forms, which are critical to their ability to cause infection. A significant virulence factor essential for pathogenicity is morphological switching, which allows *Candida albicans* to transition from its yeast form to hyphal form. This transition is regulated by key genes such as EFG1, which plays a central role in hyphal development and virulence. Some proteins and factors enhance the adhesion of *C. albicans* to breach the skin and lead to infections (21, 22). Common fungal infections include nail and skin issues such as ringworm, caused by dermatophytes like *Trichophyton rubrum*, with symptoms including peeling, cracking, redness, blistering, and itching (23, 24).

To evade immune response, fungi use a variety of strategies to survive. These include altering their outer layer, utilizing structures like glycoproteins or protective capsules, and forming biofilms. In *C. albicans*, the formation of biofilm is regulated by quorum-sensing molecules, such as farnesol. Farnesol modulates cell signaling, suppresses filamentation, and influences both virulence and immune evasion. Additionally, the ability to switch between filamentous and yeast forms is another survival tactic employed by these fungi (11).

## 4. Botany and Chemistry of Allium Species with Antifungal Components

### 4.1. Botany

In 1753, Carl Linnaeus described the *Allium* species for the first time. The name *Allium* is derived from the Greek word "allion", meaning "garlic", which reflects its long-standing use. The *Allium* genus has more than 900 species, making it one of the largest plant genera. In the kingdom of Plantae, they belong to the Amaryllidaceae family, which is a subgroup of Allioideae (25-27).

This genus is characterized by bulbs covered in membranous or fibrous tunics and ranges in height from 5 to 150 cm, with flowers forming an umbrella-like structure atop a leafless stem. In floral displays, tepals vary in color and are classified into six categories: Blue, purple, pink, red, white, and yellow. Their petals are mostly free, and they enhance the diverse vegetation in steppes, mountains, and semi-deserts (28-30).

*Allium ascalonicum*, *A. ampeloprasum*, *A. schoenoprasum*, *A. tuberosum*, *A. sativum*, and *A. cepa* are all categorized as *Allium* vegetables. These plants are believed to have originated in the Turanian-Iranian region (e.g., the center of diversity in Central Asia). These vegetables are integrated into people’s diets. For centuries, the *Allium* genus has been valued not only for its distinctive flavors and aromas but also for its medicinal properties, including its benefits against fungal infections (31-33). This genus is a source of several chemical groups, including organosulfur compounds, polyphenols and flavonoids, proteins and amino acids, saponins, alkaloids, cardenolides, vitamins, fatty acids, carbohydrates, minerals, and dietary fibers, which have medicinal benefits (34-39).

### 4.2. Chemical Classification and Biosynthetic Pathway

Secondary metabolites are small organic compounds derived from primary metabolites. Their use as medicinal or toxic agents dates back to approximately 2600 BC. Their chemical composition varies by species, making them notable for their structural diversity and potential as medicine (40).

#### 4.2.1. Sulfur Compounds

**Figure 2.**
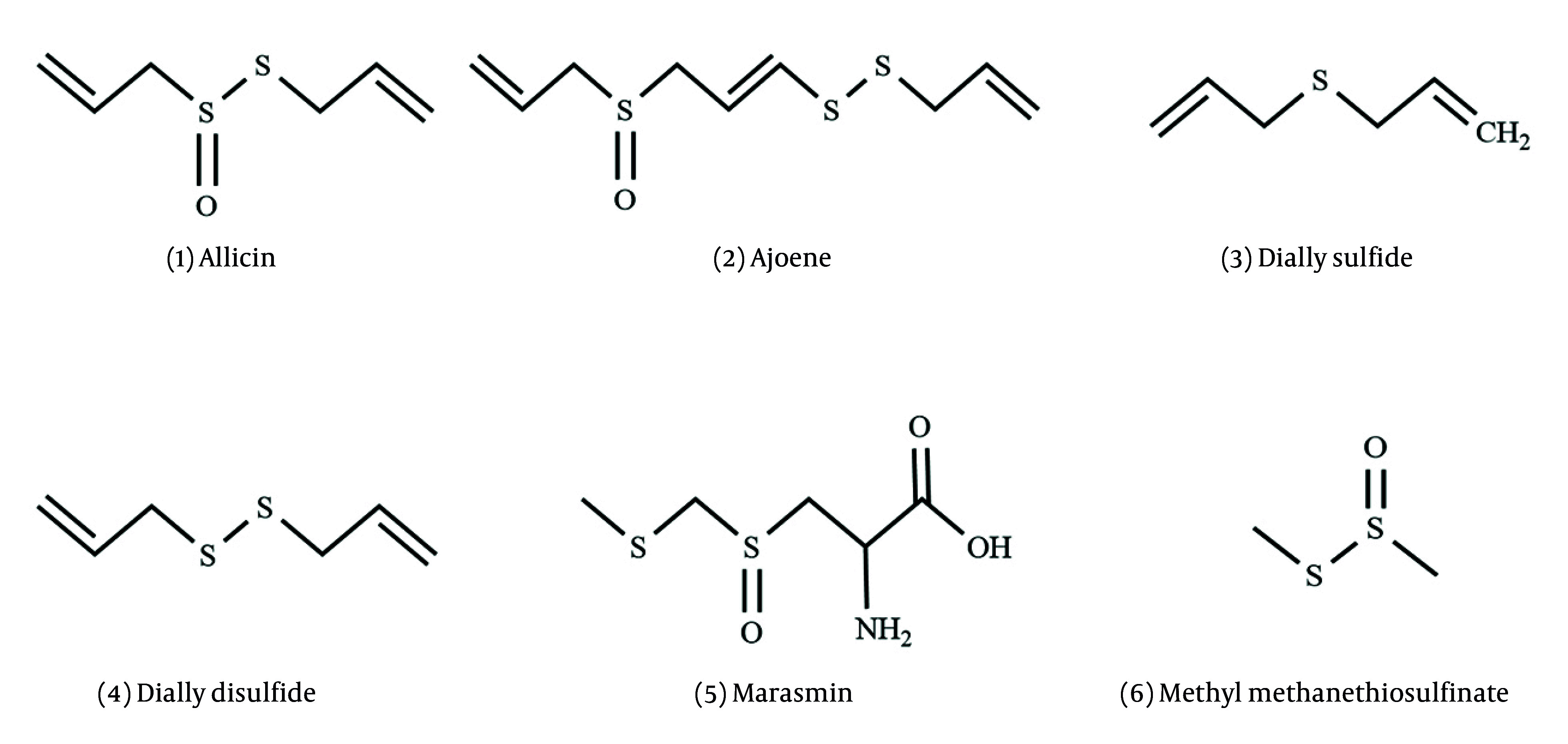
Chemical structures of sulfur compounds. (1) Allicin: Diallyl thiosulfinate; (2) ajoene: (E, Z)-4,5,9-trithiadodeca-1,6,11-triene 9-oxide; (3) diallyl sulfide: 3-(Prop-2-en-1-yl) sulfanylprop-1-ene; (4) diallyl disulfide: 3-[(Prop-2-en-1-yl) disulfanyl] prop-1-ene; (5) marasmin: (S)-2-amino-3-[(S)-2-propenylsulfinyl] propanoic acid; (6) methyl methanethiosulfinate: Methanesulfinothioic acid S-methyl ester.

The biosynthesis of organosulfur compounds begins with L-cysteine, which reacts with L-glutamic acid. The new molecule reacts with 2-propenyl carboxylic acid. This process involves several steps: Decarboxylation, oxidation, and isomerization, which result in the formation of alliin. Alliin then undergoes enzymatic catalysis by the alliinase, producing a sulfenic acid intermediate. This intermediate reacts with water to create allicin. Allicin is unstable and decomposes to produce a variety of sulfur compounds, including ajoene, methyl allyl sulfide, methyl allyl disulfide (MADS), and other sulfur compounds (41, 42). Figure 2 shows the structure of the antifungal compounds (1 - 6) that they extracted and elucidated from phytochemical studies.

In 1893, Louis Pasteur documented garlic’s antimicrobial properties when he added garlic extract to a culture container and noticed that all the bacteria were destroyed (43). In 1944, allicin (compound 1) was identified by Cavallito and Bailey, who named it allicin, as the first sulfur compound in garlic (44). The increased sensitivity of medical yeast to compound 1 highlights its potential in treating fungal infections. Pure allicin from garlic has anti-candidal effects and inhibits the growth of various fungal spores and filaments development. Allicin disrupts yeast metabolism by targeting thioredoxin reductase, impairing oxidative stress response, and stimulating cell death (45).

In 1987, ajoene (compound 2), extracted from garlic, effectively inhibits both *Colletotrichum* species and *Fusarium* species. Additionally, compound 2 is a more potent antifungal agent than compound 1 in the culture of *Aspergillus niger* and *C. albicans*. Also, ajoene exhibits antifungal activity against *Scedosporium prolificans* (46-49). Compound 1 in *A. sativum* for. pekinense bulbs, extracted and showed more activity against *Trichophyton* species than *A. cepa* and *Allium fistulosum* oils (50). Compound 2 increases the efficacy of sulfamethoxazole/trimethoprim in mice that are infected with *Paracoccidioides brasiliensis*. It promotes Th1 cytokines that increase IFN-γ and IL-12. This process stimulates a proinflammatory response (51).

*Allium suworowii* has marasmin (compound 5) as the marasmicin precursor. Marasmicin is known in South Africa due to its antifungal properties. *Allium stipitatum* and *A. altissimum* also have marasmin (52). The antifungal activity of *A. usrinum* flower extract, which contains compound 1, shows its ability to inhibit the growth of *Botrytis cinerea*, *A. niger*, and other various fungal species (53). Compound 6 was characterized by using GC/MS as a major compound in the essential oil of the fresh bulbs of *A. roseum* var. *grandiflorum*. It has highly effective antifungal activities (54).

Many phytochemical studies have shown that other members of the *Allium* genus contain organosulfur compounds. Six new organosulfur compounds and dithiosulfinate with antifungal properties were isolated from *A. sativum* var *Voghiera* from Italy (13). In Serbia, diallyl trisulfide, compound 4, and allylmethyltrisulfide were identified in garlic oil (55). In *A.* *sativum* ethanolic extracts, 1-propenyl methyl disulfide and allyl trisulfide with anticandidal activities were extracted (56). The *Allium hooshidaryae* essential oil was analyzed, and methyl disulfide and bis-methyl disulfide with moderate potency against *C. albicans* were reported (57). Diallyl trisulfide, compounds 3, and 4 are the main compounds found in garlic oil, which have shown antifungal activities against two wood-rotting fungi, *Trametes hirsuta* and *Laetiporus sulphureus*. Diallyl trisulfide was the most effective. Additionally, compound 3, isolated from garlic essential oil, shows antifungal properties against *Phytophthora nicotianae* (58, 59).

Onion extract reveals antifungal activity and inhibits the growth of various fungal pathogens due to its bioactive compounds. The major components include compound 3 and diallyl trisulfide. Allicin is also effective against zygomycete fungi, which are major contributors to mycoses. Its effectiveness varies based on spore concentration, highlighting its potential for infections in the nasopharynx through inhalation (60, 61). In Table 1, the minimum inhibitory concentration of sulfur compounds is shown.

**Table 1.** Antifungal Activities of Key Sulfur Compounds in *Allium* Species

Compounds	Sources	Parts of Plants	Fungi	Fungi Strain	Assay	MIC (µg/mL)	Ref
**(1) Allicin**	*Allium* *sativum*	Bulbs	*Aspergillus niger*	ATCC 16404	SB	30.9	(46)
			*Candida albicans*	ATCC 10231	SB	17.3	
	*A. sativum*	Bulbs	*C. parapsilosis*	Clinical strain	BM	0.15	(45)
			*C. albicans*	Clinical strain	BM	0.3	
			*Cryptococcus neoformans*	Clinical strain	BM	0.3	
			*C. tropicalis*	Clinical strain	BM	0.3	
			*C. krusei*	Clinical strain	BM	0.3	
			*Torulopsis glabrata*	Clinical strain	BM	0.3	
			*C. albicans*	Clinical strain	BM	0.8	
			*T. glabrata*	Clinical strain	BM	1.9	
	*A. sativum* for. pekinense, *A.* *cepa*, and *A. fistulosum*	Bulbs, bulbs, and whole plant, respectively	*Trichophyton erinacei*	KCCM 60411	BM	16	(50)
			*T. soudanense*	KCCM 60448	BM	16	
			*T. rubrum*	ATCC 6345	BM	32	
	*A.* *ursinum*	Flowers	*A. niger*	Field isolated	AD	100	(53)
			*Botrytis cinerea*	Field isolated	AD	60	
			*B. paeoniae*	Field isolated	AD	70	
			*Fusarium oxysporum* f.sp. *tulipae*	Field isolated	AD	140	
			*Penicillium gladioli*	Field isolated	AD	90	
			*Sclerotinia sclerotiorum*	Field isolated	AD	60	
	*A. ursinum*	Leaves	*A. niger*	Field isolated	AD	120	(53)
			*B. cinerae*	Field isolated	AD	80	
			*B. paeoniae*	Field isolated	AD	100	
			*F. oxysporum* f.sp. *tulipae*	Field isolated	AD	160	
			*P. gladioli*	Field isolated	AD	120	
			*S. sclerotiorum*	Field isolated	AD	80	
**(2) Ajoene**	*A. sativum*	Bulbs	*C. albicans*	ATCC 10231	SB	7.6	(46)
			*A. niger*	ATCC 16404	SB	16.6	
	*A. sativum*	Bulbs	*F. oxysporum*	Field isolated	SG	25	(47)
			*F. incarnatum*	Field isolated	SG	25	
			*F. udum*	Field isolated	SG	25	
			*Colletotrichum* sp.	Field isolated	SG	25	
			F. lini	Field isolated	SG	100	
			*Alternaria triticina*	Field isolated	SG	100	
	*A. sativum*	Cloves	*Scedosporium prolificans*	Clinical strain	BM	> 8	(49)
	Commercial	-	*Paracoccidioides brasiliensis*	Pb 18	BM	> 50	(51)
**(3) Dially sulfide**	Commercial	-	*T. erinacei*	KCCM 60411	BM	128	(50)
			*T. soudanense*	KCCM 60448	BM	128	
			*T. rubrum*	ATCC 6345	BM	> 128	
**(4) Dially disulfide**	Commercial	-	*T. erinacei*	KCCM 60411	BM	8	(50)
			*T. soudanense*	KCCM 60448	BM	16	
			*T. rubrum*	ATCC 6345	BM	16	
**(6) Methyl methanethiosulfinate**	*A.* *roseum*	Bulbs	*C. albicans*	ATCC 10231	BM	19	(54)

Abbreviations: MIC, minimum inhibitory concentration; SB, sabouraud broth; BM, broth microdilution; AD, agar dilution; SG, spore germination.

#### 4.2.2. Polyphenols and Flavonoids

**Figure 3.**
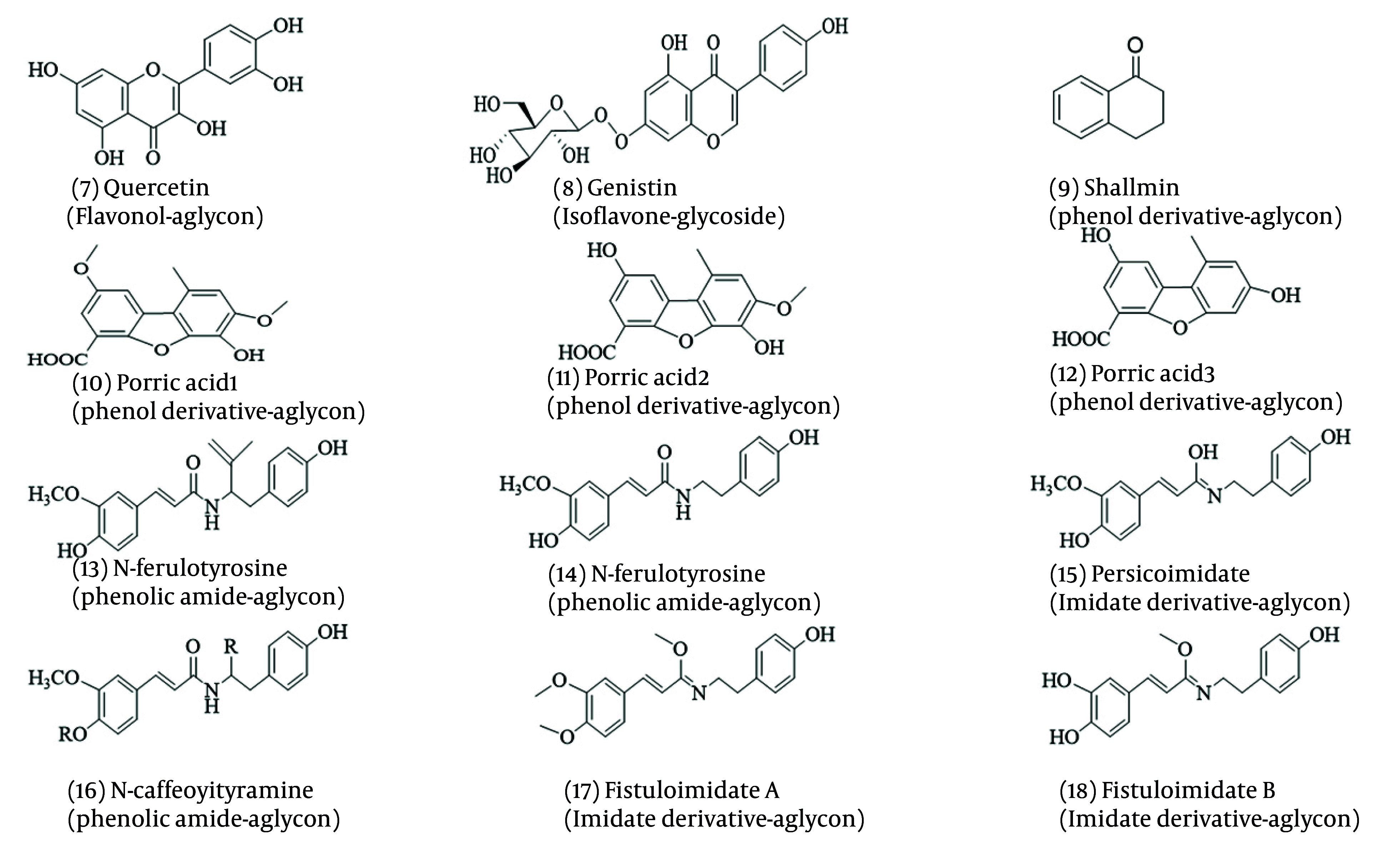
Chemical structures of polyphenol compounds

Phenolic compounds are synthesized through the phenylpropanoid pathway using phenylalanine and tyrosine amino acids, followed by the Shikimate pathway (62-64). *Allium* vegetables are notable for their high content of phenolic compounds, particularly polyphenols, flavonoids, and anthocyanins (65). *Allium*’s polyphenol derivatives extracted and elucidated with antifungal activities are shown in Figure 3. 

Three new dibenzofurans, compounds 10, 11, and 12, have been identified from *Allium porrum*. Additionally, compounds 13 and 14 extracted from *A. porrum* and *A. sativum* demonstrated antifungal efficacy against *F. culmorum* with a minimum inhibitory concentration of 22 µg/mL (66, 67). Compounds 7 and 8 have been used to treat fungal infections. Recent studies have increasingly focused on their properties against infectious diseases (68, 69). Compounds 14, 15, and 16 were isolated from Persian leek and evaluated for their antifungal activities. They were inhibited by *Penicillium italicum*, *A. niger*, and *B. cinerea*, highlighting their potential role in plants’ defense against pathogens. Similar compounds have also been identified in *A. tripedale* (70, 71).

Persian shallot contains compound 9, which exhibits fungistatic and fungicidal effects on a wide range of fungi, including *T. rubrum*, *C. albicans*, *F. oxysporum*, *Saccharomyces cerevisiae*, and *A. niger* (72, 73). Compounds 17 and 18, extracted from *A. fistulosum*, have been shown to possess antibacterial and cytotoxic properties (74). Additionally, flavonols derived from *A. cepa* have been tested as bio-fungicides against *Ceracospora arachidicola* and *Cercosporidium personatum* (75).

#### 4.2.3. Saponins and Steroids

**Figure 4.**
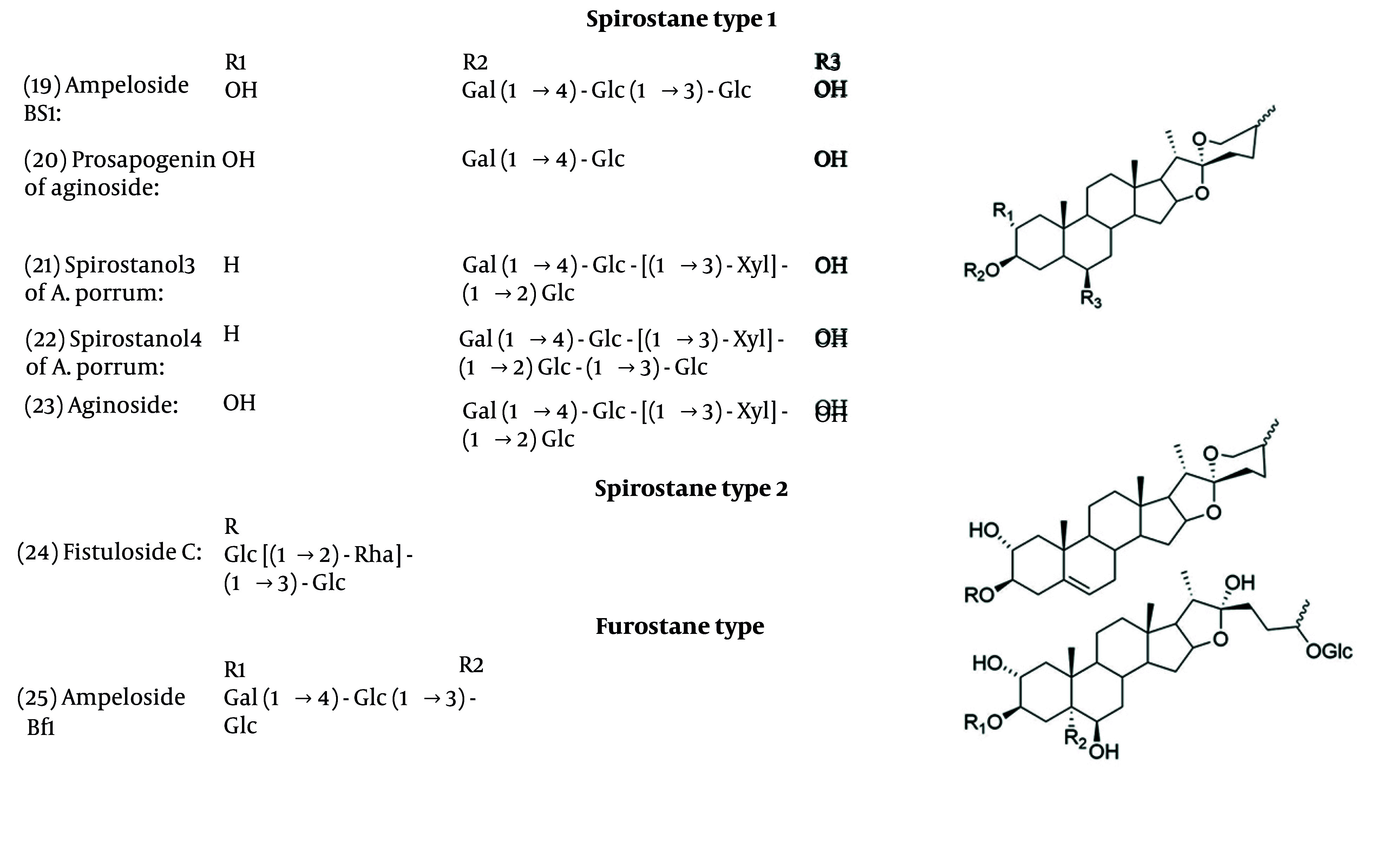
Chemical structure of saponins

Saponins’ backbones are established on three types: Furostane, spirostane, and cholestane. Their biosynthesis can be separated into three main steps. First, involves the production of 2,3-oxidosqualene via the mevalonate and then 2C-methyl erythritol 4-phosphate pathways. In the second step, β-sitosterol and cholesterol were made from catalytic reactions. The last step involves the formation of steroidal saponins at different side chain positions, C-16, C-26, and C-26 (41, 76). The structures of isolated saponins with antifungal activities are shown in Figure 4. 

Several *Allium* species have been found to contain saponins with antifungal properties. Research on *A. ampeloprasum* bulbs led to the discovery of compounds 19, 20, and 25, which demonstrated activity against fungi such as *C. albicans* and *A. niger* (77). In *A. porrum*, 21 and 22 were identified and found to be active against the fungus *F. culmorum*, with some showing additional antiproliferative effects (78).

Many saponins with varied structures, including furostane, spirostane, cholestane, and oleane types, were identified and exhibited antifungal activity against *F. culmorum* and *C. albicans* with effective concentrations ranging between 25 and 100 μg/mL (79, 80). *Allium fistulosum* (Welsh onion) contains saponins such as Fistuloside A, B, and compound 24, with compound 24 showing the highest antifungal activity (81). Minutoside A, B, and C were identified in *A. minutiflorum*, with minutoside B being the most potent against fungal pathogens (82).

The antifungal effects of compounds Voghieroside A to E and compound “Saponin 6” from *A. sativum* var. *Voghiera* were tested against various fungal pathogens. The “Saponin 6” emerged as the most effective among them (83). In *A. cepa*, compounds ceposide A-C showed enhanced antifungal effects, especially when used together against pathogens like B. cinerea and *Trichoderma atroviride* (84).

Through an antifungal study, compounds 21, 22, and 23 inhibited *C. albicans* (85). Additionally, the compound 23 from *A. nigrum* also exhibited activity against various multiple soil and airborne fungal pathogens (86). Seeds of Persian leek (*A. porrum*) yielded persicosides A to E. Persicoside A and B, which were particularly effective against *P. italicum* and *A. niger*, indicating that the spirostane structure contributes to antifungal efficacy (87). In Table 2, the minimum inhibitory concentrations of saponins are shown.

**Table 2.** Antifungal Activities of Saponins

Compounds	Sources	Parts of Plants	Fungi	Fungi Strain	Assay	MIC (µg/mL)	Ref
**(19) Ampeloside Bs1**	*Allium ampeloprasum*	Bulbs	*Candida albicans*	ATCC 10231	SB	100	(77)
			*Aspergillus niger*	ATCC 16404	SB	400	
**(20) Prosapogenin of aginoside**	*A. ampeloprasum*	Bulbs	*C. albicans*	ATCC 10231	SB	100	(77)
			*A. niger*	ATCC 16404	SB	> 400	
**(21) Spirostanol 3 of leek**	*A. porrum*	Bulbs	*C. albicans*	DAY 185	IFG	5.8	(85)
**(22) Spirostanol 4 of leek**	*A. porrum*	Bulbs	*C. albicans*	DAY 185	IFG	13.3	(85)
**(23) Aginoside**	NR	NR	*C. albicans*	DAY 185	IFG	47	(85)
**(24) Fistulosides C**	*A.* *fistulosum*	Edible parts	*Saccharomyces cerevisiae*	IFO 0233	SB	3.1	(81)
			*C. albicans*	ATCC 10231	SB	6.1	
**(25) Ampeloside Bf1 **	*A.* *ampeloprasum*	Bulbs	*C. albicans*	ATCC 10231	SB	> 800	(77)
			*A. niger*	ATCC 16404	SB	> 800	

Abbreviations: NR, not reported; SB, sabouraud broth; IFG, inhibition of fungal growth.

#### 4.2.4. Miscellaneous Compounds

**Figure 5.**
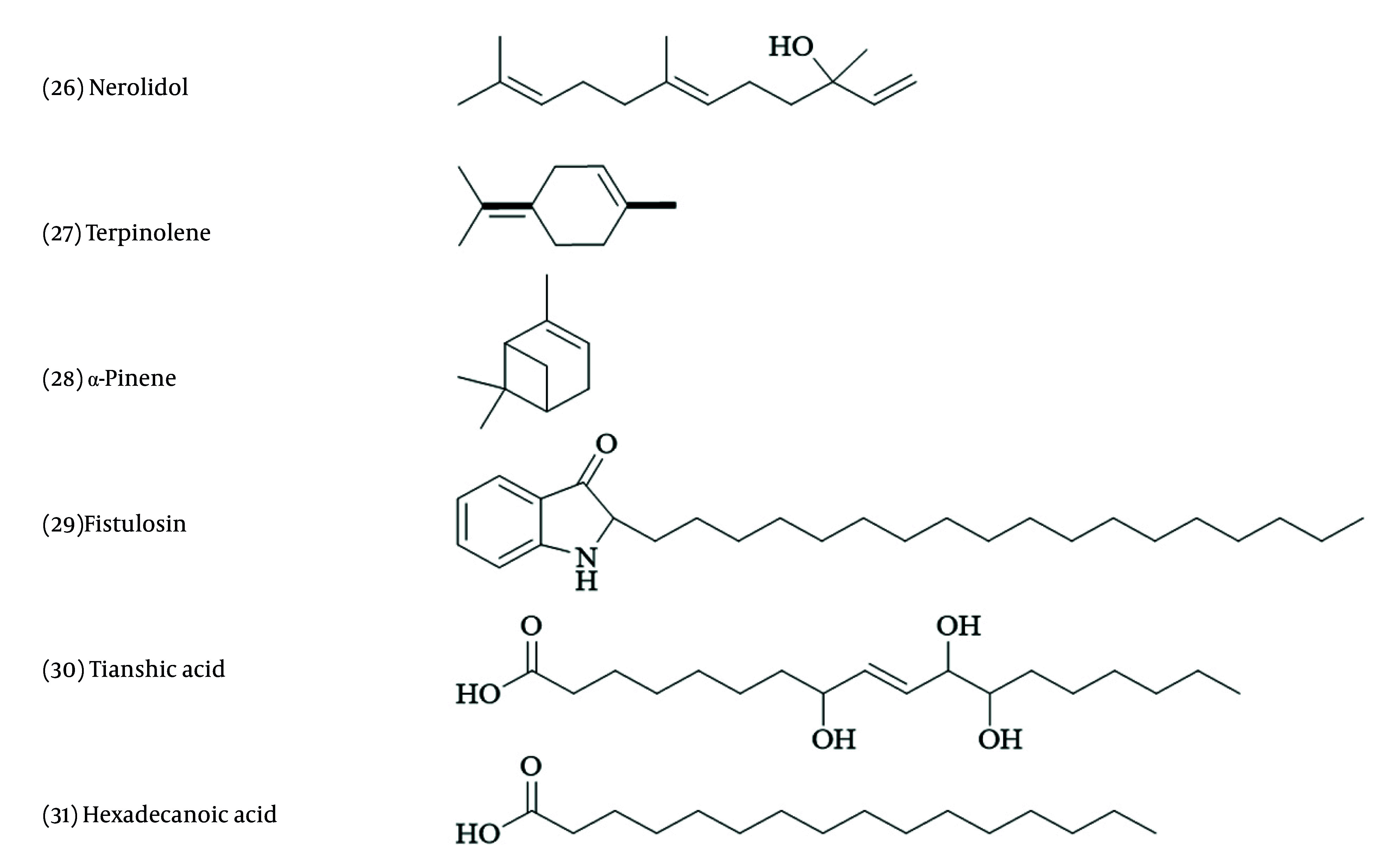
Chemical structures of miscellaneous compounds

Miscellaneous terpenoids and amino acids were isolated from *Allium* species (67). When comparing the level of terpene compound production of *A. sativum* infected with the white rot disease pathogen to those of healthy samples cultivated in vitro, it was observed that antifungal terpene synthesis increased in the infected samples. Among various terpenes, nerolidol and terpinolene (Figure 5) inhibited the growth of *Sclerotium cepivorum*, while α-pinene had the opposite effect (88).

Compound 29, derived from *A. fistulosum* roots, has shown strong antifungal action on *F. oxysporum* by inhibiting the process of protein synthesis (89). An antifungal protein from *A. tuberosum* shows similarities to chitinase, demonstrating inhibitory activities on several fungi, such as *B. cinerea* (90).

Allivin, a new protein from garlic bulbs, ascalin from shallot bulbs, and allicepin from onion inhibited the growth of *B. cinerea* (91-93). Another antifungal peptide, named NpRS, with the same antifungal activity, and a protein named alliumin, which displayed antifungal activity on *Mycosphaerella arachidicola*, were isolated from garlic (94, 95). *Allium sativum* agglutinin, a lectin (~14kDa) from garlic bulbs, has demonstrated considerable therapeutic potential, including anticancer, antimicrobial, antifungal (especially against *Candida* species), and other biological effects (96).

Fatty acids are chemical groups isolated from *Allium* species. For example, a monoacylglyceride containing an unsaturated fatty acid with a Δ9 double bond, and compound 30, both isolated from *A. fistulosum* seeds, exhibited growth inhibition effects on *Phytophthora capsica*, a known plant pathogen (97). Compound 31 is the major component in *A. roseum* essence. This essential oil inhibited *Fusarium solani* and *B. cinerea* (98)). In *A. cepa*, quercetin and kaempferol have shown antibacterial effects against *Bacillus cereus*, *Escherichia coli*, *Pseudomonas aeruginosa*, and *Staphylococcus aureus* (68, 69).

## 5. Antifungal Mechanisms and Structure-Activity Relationship Studies

Briefly, organosulfur compounds inhibit enzymes crucial for metabolism, disrupting cellular function by compromising the structural integrity and functionality of fungal cells, affecting membrane permeability, or disrupting cellular processes. They induce oxidative stress, damaging amino acids and DNA. Moreover, they exhibit quorum-sensing inhibitory effects, which prevent bacterial communication that coordinates biofilm formation and virulence, making them more susceptible to antimicrobials and the immune response (99-101). Organosulfur compounds have thiol reactivity by targeting thiol groups in proteins and enzymes (102). The presence of free thiol and disulfide bonds in these compounds enhances their potency. For example, allicin, which is a thiosulfinate containing a reactive sulfur group, exhibits a minimum inhibitory concentration of 0.05 µg/mL against *C. albicans*. In contrast, its oxidized sulfoxide analogs require concentrations greater than 100 µg/mL to achieve a similar level of inhibition (103, 104).

Phenolic compounds and flavonoids inhibit fungal growth by a variety of mechanisms. They penetrate cells and disrupt key metabolic processes, including the synthesis of ergosterol, chitin, glucan, RNA, proteins, and glucosamine. Also, they can change the function of mitochondria, efflux pumps, and inhibit the fungal biofilms (14, 66, 105, 106). These effects increase with the double bonds between C2 and C3, enhancing the binding to fungal enzymes (e.g., flavone is stronger than flavanone) (107). Increasing the number of hydroxyl (OH) groups can improve the antifungal effect (quercetin, with a 3 OH group on its C-ring, is more potent than kaempferol, which features a 4 oxo group). Additionally, aglycone compounds penetrate cell membranes more effectively than glycosides. However, methylation (O-CH3) at C3 reduces the antifungal effect (108-110).

Steroidal saponins’ antifungal activities are performed through several mechanisms that induce damage to the plasma membrane, disrupt cell membrane functions, and enhance membrane permeability (111, 112). They bind to sterols in membranes, disrupt the membrane structure, and lead to cell collapse (113). Steroidal saponins with a spirostanol backbone are more potent than furostanol; for example, persicoside A (spirostanol) in Persian leek is more effective than persicoside C (furostanol) (87). Aglycone form and OH groups in C3 and C6 increase the effects, whereas C-5 oxygenation and excess sugar potency reduce them. Sugar chain length affects water solubility; monosaccharides reduce water solubility, while trisaccharides increase it, but reduce the compound’s ability to cross cell membranes (41, 87, 114).

Terpenoids act by damaging the cell membrane of pathogens and impairing mitochondrial processes, leading to disruption of electron transport and inhibition of ATPase activity, resulting in cell death (115, 116). Terpenoids’ antifungal activities increase in the presence of carbonyl (C=O) groups (117), and OH groups (as demonstrated by the lower minimum inhibitory concentration of terpinen-4-ol compared to α pinene against *C. albicans*, indicating enhanced potency due to the OH group) (118, 119). The bicyclic backbone is more effective than those with a monocyclic structure (120). While the glycoside structure decreases the effects rather than the aglycone (121).

## 6. Discussion

The antifungal characteristics of *Allium* species have become a focal point of research due to their potential as natural therapeutic agents. Various bioactive constituents, including organosulfur compounds (allicin and ajoene) (46, 47), polyphenols and flavonoids (quercetin and cinnamic acid derivatives) (68, 70), steroidal saponins (spirostane and furostane type) (87), and miscellaneous compounds (proteins and terpenes), play a significant role in their antifungal activities (88, 91). They exhibit various mechanisms, including disrupting the cell membrane, penetrating cells, inducing oxidative stress, inhibiting enzymatic and metabolic processes, changing the functions of essential organelles (e.g., efflux pumps), binding to membrane constituents and collapsing cells, and disrupting electron transport and inhibiting ATPase activities in mitochondria (14, 101, 116). These mechanisms are especially effective in combating clinical pathogens such as *Candida* and *Aspergillus* species (122).

The potential applications of *Allium*’s antifungal properties are extensive, spanning human health, agriculture, and food preservation. In healthcare, *Allium* extracts, especially garlic, have been studied as treatments for both superficial and systemic fungal infections. An important advantage of using *Allium*-based treatments is the reduced likelihood of inducing drug resistance. The complex array of bioactive compounds in *Allium* may target multiple pathways in fungal cells, making it more challenging for fungi to develop resistance. Allicin and ajoene affect fungi by targeting enzymes, damaging membranes, and disrupting stress responses. Additionally, allicin enhances fungal sensitivity to treatments by inhibiting biofilm growth and decreasing ergosterol levels, which is vital for resistant strains. *Allium*-derived compounds may be beneficial as adjunct therapies for systemic infections, particularly in individuals at risk for developing antifungal resistance. This characteristic is particularly relevant given the rise of resistant strains such as *Candida auris* (first identified in 2009 during an outbreak in Japan), which pose significant challenges in clinical settings. Additionally, *Allium* compounds, particularly sulfur compounds and flavonoids, may work together or with other natural products to enhance antifungal effectiveness, especially against resistant strains (47, 106, 122-124).

In agricultural and food processing contexts, *Allium* extracts can serve as biopesticides to combat fungal diseases affecting crops. Utilizing these natural fungicides presents an environmentally friendly alternative to synthetic counterparts. For example, garlic oil emulsions demonstrated a significant reduction of up to 50% in *B. cinerea* lesions on strawberry flowers, with treated plants yielding 27% more fruit compared to untreated, which demonstrates garlic’s role as a biopesticide in fruit crops (31, 32, 125, 126). Compounds from *Allium* species represent promising natural agents for food preservation, as they effectively prevent fungal contamination in fruits, vegetables, and processed products. This antifungal activity can prevent the growth of foodborne fungi such as *Aspergillus flavus*, which produces harmful aflatoxins. This application could improve the safety and longevity of processed foods without resorting to synthetic chemical preservatives (31, 32, 125).

A key limitation is the compositional variability among *Allium* extracts, with plant variety, cultivation environment, extraction methodology, and storage conditions all influencing the levels of active compounds such as allicin. This inconsistency presents challenges in standardizing dosages for therapeutic or agricultural applications, complicating the ability to achieve reliable results. Additionally, the stability of *Allium* compounds raises concerns. For instance, allicin is unstable and can degrade quickly when exposed to heat, light, or oxygen in biological environments due to the presence of a thiosulfinate group (R-S(=O)-S-R'). It makes it more electrophilic and highly reactive. It can also interact with thiol groups in proteins, leading to the formation of mixed disulfides or sulfenic acids. This instability limits its shelf life and effectiveness compared to more stable synthetic antifungal agents. While in vitro studies exhibited the antifungal efficacy of *Allium* extracts against a range of fungi, there is a notable lack of comprehensive in vivo clinical trials confirming their effectiveness in human populations. Therefore, research is needed for further investigation into formulation strategies and delivery systems (nanoencapsulation or prodrug) that can stabilize allicin and enhance its therapeutic potential (42, 127, 128).

Synergistic investigations combining *Allium* extracts with conventional antifungal agents may provide novel approaches to overcome resistant fungal infections, while reducing drug dosage and associated side effects. When *Allium* compounds are combined with synthetic antifungal drugs like amphotericin B, they can sensitize resistant strains like *Candida* species to treatment by increasing drug uptake, inhibiting efflux pumps, and decreasing ergosterol levels in the membrane (129).

### 6.1. Conclusions

This review synthesizes biomolecular data on *Allium*-derived antifungals, spotlighting underutilized saponins and the need for improved allicin formulations. *Allium* species such as garlic, onions, and leeks have a long-standing history in human culture and are rich in bioactive components, including steroidal saponins, organosulfur compounds, phenolics, and peptides. These substances exhibit a wide array of biological activities, particularly antifungal activity. With the growing challenge of drug resistance in fungal infections, there is an urgent demand for new treatments that are effective and have lower toxicity. *Alliums* offer a promising opportunity for the discovery and isolation of new antifungal compounds. This process involves discovering new molecules, modifying existing structures, and examining their structure-activity relationships, mechanisms of action, and potential synergistic effects. Further clinical trials are required to confirm the antifungal efficacy of *Allium* species in humans. Such studies should address both topical and systemic applications, with careful evaluation of safety, therapeutic effectiveness, and long-term outcomes. Integrating traditional knowledge with modern scientific approaches underscores the importance of *Allium* not only as a dietary component but also as a valuable resource for promoting health and preventing disease. Continued research into chemical constituents and therapeutic potential remains essential for developing innovative strategies to combat fungal infections and improve overall well-being. By emphasizing the roles of active compounds, we can fully leverage the health benefits of *Allium*, facilitating advancements in functional foods and natural medicinal treatments. We recommend standardized extract characterization and rigorous clinical studies to translate these insights into effective antifungal therapies.
